# Relationship between multi-nutrient intake and bone loss and osteoporosis in U.S. adults: Findings from NHANES

**DOI:** 10.1097/MD.0000000000040768

**Published:** 2024-12-13

**Authors:** Wenfeng Hu, Xiaotong Feng, Chaoqun Wen

**Affiliations:** a Department of Orthopedics, Dongguan Hospital of Traditional Chinese Medicine, Dongguan, China; b Emergency Department, Zhuhai Third People’s Hospital, Zhuhai, Guangdong Province, China; c Department of Orthopedics, The Fifth Affiliated Hospital of SunYat-Sen University, Zhuhai, Guangdong Province, China.

**Keywords:** bone mineral density, dietary potassium, magnesium intake, NHANES, osteopenia, osteoporosis, sodium

## Abstract

The relationship between dietary nutrient intake and bone mineral density (BMD) has not been clarified. In the U.S. population, we have demonstrated that dietary intake of multiple nutrients (potassium, magnesium, and sodium) is positively associated with BMD and negatively associated with the prevalence of osteopenia. This study examined whether there is an association between dietary potassium, magnesium, and sodium intake and BMD, osteopenia, and osteoporosis, using data from the National Health and Nutrition Examination Surveys from 2005 to 2010, 2013 to 2014, and 2017 to 2018. We assessed the association of dietary potassium, magnesium, and sodium intake with BMD in 10,355 National Health and Nutrition Examination Survey participants during 2005 to 2010, 2013 to 2014, and 2017 to 2018. BMD of the whole femur was estimated by dual-energy X-ray absorptiometry. We utilized multiple linear regression models to examine the associations of dietary potassium, magnesium, and sodium intake with femoral BMD, osteopenia, and osteoporosis, after adjusting for various confounders. Dietary potassium, magnesium, and sodium intake are positively correlated with femur BMD when corrected for the confounders of age, sex, race/ethnicity, smoking behavior, education level, body mass index, poverty income ratio, serum uric acid, serum cholesterol, potential renal acid load, dietary calcium intake, dietary protein intake, and dietary vitamin D intake. Dietary intake of potassium, magnesium, and sodium was adversely correlated with the development of osteopenia and osteoporosis. Our study showed that intake of dietary nutrients (potassium, magnesium, and sodium) was correlated positively to femur BMD and adversely to osteopenia and osteoporosis in the U.S. population. Further research is needed on the association of dietary elemental intake with BMD.

## 1. Introduction

Osteoporosis is a prevalent generalized bone disease that leads to bone weakness and an elevated risk of fracture.^[1]^ Osteoporosis is also considered to be a silent disease because there are usually no symptoms until the first fracture occurs.^[2]^ Osteoporosis-induced fractures are becoming more prevalent in females beyond the age of 55 and in males beyond the age of 65, resulting in a large number of skeletal-related illnesses, increasing the mortality rate and the cost of medical services.^[3]^ Osteoporosis is a globally significant public health concern, impacting over 200 million people around the world and 54 million seniors in the U.S.^[4]^ In the United States, the cost of treating osteoporosis-related fractures annually equals or exceeds the cost of treatment for cardiac strokes, breast cancer, or cerebrovascular accidents.^[5,6]^ Prior research has shown that dietary potassium intake is positively associated with bone mineral density (BMD), yet fewer studies have explored the association of dietary sodium and magnesium with BMD, and the results have been conflicting.^[7–9]^

Dietary nutritional factors are particularly essential for skeletal integrity, and it is generally recognized that calcium and vitamin D are important nutrients for skeletal integrity.^[10,11]^ Yet, the effects of other nutrients on bone are poorly understood, and it is worth noting that a calcium-rich diet usually contains many other essential nutrients for bone health, including potassium, sodium, magnesium, phosphorus, and protein.^[8,12]^ Dietary nutrients can influence BMD by interacting with underlying elements, for example, alkaline elements can improve bone health by neutralizing acid load and regulating acid–base balance.^[7,13]^ In 2016, SH Kong et al found that dietary potassium intake was beneficial for bone health in populations with lower intakes of calcium.^[10]^ However, a few studies have examined the association among dietary potassium, sodium, and magnesium intake and BMD in normal populations, and the results have been inconsistent. Katherine L Tucker et al showed through a food-frequency questionnaire that higher intakes of potassium and magnesium were related to a smaller loss of hip BMD in an older population.^[10]^ In contrast, another prospective cohort study identified an increase in magnesium intake slightly above the recommended dietary intake as being associated with an increase in lower arm and wrist fractures.^[14]^ Another cross-sectional study of 330 8-year-old children found negative or no association with the dietary intake of potassium and magnesium with BMD.^[15]^

In summary, there is limited evidence regarding the association of dietary intake of potassium, sodium, and magnesium with osteoporosis. Therefore, this study performed a full-sample cross-sectional research to explore the association of dietary nutrient intake and BMD in the American people, which was conducted on the basis of NHANES data from 2005 to 2010, 2013 to 2014, and 2017 to 2018. This is likely to be able to provide dietary nutritional input recommendations for people with osteoporosis.

## 2. Materials and methods

### 2.1. Study population

**Figure 1.**
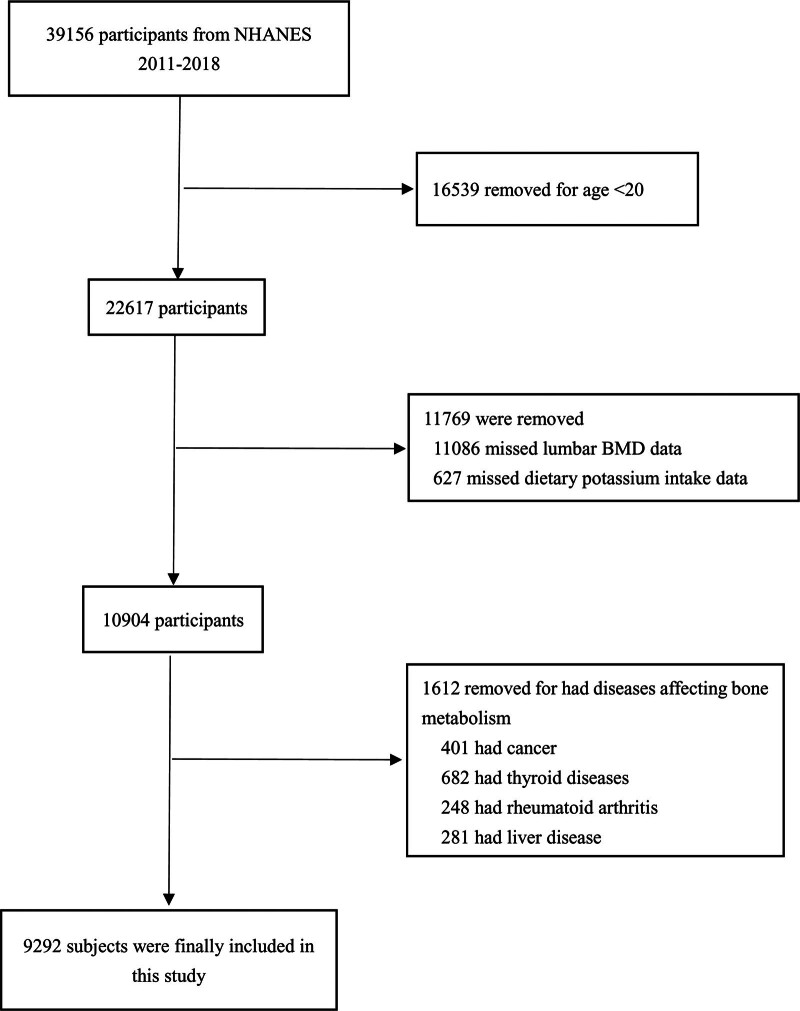
Flowchart of participants’ selection.

The data in this study come from the NHANES 2005 to 2010, 2013 to 2014, and 2017 to 2018 surveys, which is a survey of a large portion of the U.S. population using a complex multi-stage stratified clustered probability sampling design. We merged the data from the NHANES 2005 to 2006, 2007 to 2008, 2009 to 2010, 2013 to 2014, and 2017 to 2018 cycles for our analysis. The approaches used for the refrigerator study were conducted in accordance with the appropriate NHANES guidance and provisions. 39,156 individuals from NHANES 2011 through 2018 were originally enrolled in this study. Subsequently, 16,539 teenagers aged < 20 years, 11,769 participants who lacked BMD or dietary elemental intake data, in addition to 1612 participants with diseases causing disturbance in the metabolism of BMD, were excluded, and 10,355 qualified participants were enrolled for the final stage of the research. The process of selecting participants is illustrated in Figure 1.

### 2.2. Assessment of macronutrients intake distribution

Dietary potassium, magnesium, and sodium intake were the exposure variables in this study. Dietary intake data are obtained by a partnership between the U.S. Department of Agriculture and the U.S. Department of Health and Human Services. Under this partnership, Department of Health and Human Services’s National Center for Health Statistics (NCHS), Health and Nutrition Examination Survey Division is responsible for all aspects of survey sample design and data collection, and U.S. Department of Agriculture’s Food Survey Research Group is responsible for dietary data collection methods, maintenance of the database used to code and process the data, and data review and processing. The dietary intake data are used to estimate the types and amounts of foods and beverages (including all types of water) consumed during the 24-hour period prior to the interview (midnight to midnight) and to estimate intakes of energy, nutrients, and other food components from those foods and beverages.

### 2.3. Assessment of BMD

BMD (measured in g/cm²) was assessed using dual-energy X-ray absorptiometry. NHANES performed dual-energy X-ray absorptiometry scans using a Hologic QDR 4500A fan beam densitometer. According to World Health Organization recommendations, BMD T-scores represent the number below or above the standard deviation of the reference group. According to World Health Organization recommendations, male and female individuals aged between 20 and 29 years were selected as the reference group for the current study because prospective data showed femoral bone loss in female participants in their 30s. Osteopenia was defined as a T-value between -1.0 and -2.5, osteoporosis was defined as less than or equal to -2.5, and a T-value greater than or equal to -1.0 was considered normal. A T-value of less than -1.0 was considered to be a low BMD, which includes osteopenia and osteoporosis.

### 2.4. Other covariates

Age, sex, race/ethnicity, smoking behavior, education, body mass index, poverty income ratio, serum uric acid, serum cholesterol, potential renal acid load (PRAL), dietary calcium intake, and dietary vitamin D intake were considered to be covariates. For more information on covariates, visit the NHANES website.

### 2.5. Statistical analysis

All the statistical analyses used in this study were conducted with the R statistical package and EmpowerStats (http://www.empowerstats.com). Participants were categorized into normal and low BMD groups based on their bone mass for description. Multiple linear regression models were used to assess the association between dietary potassium, sodium, and magnesium intake and femur BMD, low BMD, and osteoporosis. Unadjusted models were created first, followed by adjusted models based on age, sex, and race (Model 2). A fully adjusted model (Model 3) was then calculated based on the variables of age, gender, race/ethnicity, smoking behavior, educational level, BMI, poverty income ratio, serum uric acid, serum cholesterol, PRAL, dietary calcium intake, dietary Vitamin D intake. The model was then calculated by age, sex, and race/ethnicity. Tests for interactions were then stratified by age, gender, and race/ethnicity. In this analysis, a *P* value of <.05 was considered statistically significant.

## 3. Results

### 3.1. Study participants and baseline characteristics

The characteristics of participants were shown in Table 1. This study included 10,355 participants aged 20 years and older. The normal and low BMD groups were similar in terms of smoking and dietary vitamin D intake, whereas age, gender, ethnicity, education, serum total cholesterol, serum uric acid, PRAL, and dietary calcium intake were significantly different between the 2 groups.

**Table 1:** Characteristics of the study population based on dietary potassium intake quartiles.

Dietary potassium intake (g)	Normal (N = 8970)	Low BMD (N = 1385)	*P* value
Age (years)	45.34 ± 15.09	54.47 ± 15.95	<.001
Gender			<.001
Male	5551 (53.61%)	337 (24.33%)	
Female	4804 (46.39%)	1048 (75.67%)	
Race/ethnicity			<.001
Mexican American	2090 (20.18%)	228 (16.46%)	
Other Hispanic	980 (9.46%)	119 (8.59%)	
Non-Hispanic White	4373 (42.23%)	696 (50.25%)	
Non-Hispanic Black	2134 (20.61%)	189 (13.65%)	
Other race: including multi-racial	778 (7.51%)	153 (11.05%)	
Education (%)			<.001
<9th grade	1184 (11.43%)	181 (13.07%)	
9–11th grade	1594 (15.39%)	229 (16.53%)	
High school graduate	2380 (22.98%)	322 (23.25%)	
College degree or above	5188 (50.10%)	649 (46.86%)	
Not reported	9 (0.09%)	4 (0.29%)	
Smoker			.030
Yes	4579 (44.22%)	631 (45.56%)	
No	5776 (55.78%)	754 (54.44%)	
PRAL			<.001
Acid load	2272 (21.94%)	396 (28.59%)	
Alkaline load	7747 (74.81%)	928 (67.00%)	
Not reported	336 (3.24%)	61 (4.40%)	
BMI (kg/m^2^)	28.49 ± 5.55	24.49 ± 4.50	<.001
PIR	2.60 ± 1.58	2.48 ± 1.54	<.001
Dietary calcium intake (mmol/L)	928.74 ± 575.46	833.29 ± 537.03	<.001
Serum cholesterol (mmol/L)	5.11 ± 1.04	5.24 ± 1.08	<.001
Serum uric acid (mmol/L)	320.49 ± 80.64	291.04 ± 77.11	<.001
Dietary Vitamin D intake	4.55 ± 4.55	4.41 ± 4.59	.014
Lumbar spine BMD (g/cm²)	1.04 ± 0.15	0.87 ± 0.12	<.001
Total femur BMD (g/cm²)	0.99 ± 0.16	0.74 ± 0.07	<.001
Femur neck BMD (g/cm²)	0.85 ± 0.15	0.64 ± 0.08	<.001

Mean ± SD for continuous variables: the *P* value was calculated by the weighted linear regression model. (%) for categorical variables: the *P* value was calculated by the weighted chi-square test.

BMD = bone mineral density, BMI = body mass index, PIR = poverty income ratio.

### 3.2. Multiple dietary nutrient intake and T-score, low BMD disorder, and osteoporosis

Tables 2 and 3 show the associations between multiple nutrient intake and T-score, low BMD disorder and osteoporosis. Potassium intake was positively associated with T-score (β = 0.141 95% CI: 0.118–0.164, *P* < .001) and was associated with a lower risk of low BMD (OR = 0.730 95% CI: 0.674–0.791, *P* < .001), and with a lower risk of osteoporosis (OR = 0.644 95% CI: 0.485–0.856, *P* = .002). Magnesium intake also positively correlated with T-score (β = 0.190 95% CI: 0.129–0.251, *P* < .001), and with reduced risk of BMD (OR = 0.624 95% CI: 0.521–0.747, *P* < .001), but was not associated with risk of developing osteoporosis (OR = 0.334 95% CI: 0.038–2.952, *P* = .324). Sodium intake also had a positive association to T-score (β = 0.062 95% CI: 0.048–0.075, *P* < .001) and was related to a lesser risk of low BMD (OR = 0.848 95% CI: 0.807–0.892, *P* < .001), but not to a risk of developing osteoporosis (OR = 0.842 95% CI: 0.704–1.007, *P* = .060).

**Table 2:** Relationship between micronutrient distribution and bone mineral density in 10,355 participants in NHANES from 2005 to 2010, 2013 to 2014, and 2017 to 2018.

	Total femur T-score β (95% CI)			LOW BMD OR (95% CI)		
	Crude	Model 1	Model 2	Crude	Model 1	Model 2
Dietary potassium intake	0.116 (0.097, 0.136) < 0.001	0.151 (0.132, 0.169) < 0.001	0.141 (0.118, 0.164) < 0.001	0.823 (0.781, 0.867) < 0.001	0.776 (0.733, 0.821) < 0.001	0.730 (0.674, 0.791) < 0.001
Q1	Reference	Reference	Reference	Reference	Reference	Reference
Q2	0.050 (-0.022, 0.121) 0.174	0.134 (0.066, 0.202) < 0.001	0.111 (0.050, 0.172) < 0.001	0.902 (0.773, 1.052) 0.188	0.792 (0.673, 0.932) 0.005	0.748 (0.625, 0.894) 0.001
Q3	0.155 (0.086, 0.225) 0.00001	0.298 (0.232, 0.364) < 0.001	0.231 (0.169, 0.294) < 0.001	0.784 (0.669, 0.919) 0.003	0.665 (0.563, 0.785) < 0.001	0.623 (0.514, 0.756) < 0.001
Q4	0.357 (0.288, 0.426) < 0.001	0.495 (0.429, 0.561) < 0.001	0.416 (0.344, 0.488) < 0.001	0.554 (0.467, 0.658) < 0.001	0.464 (0.387, 0.555) < 0.001	0.411 (0.324, 0.523) < 0.001
*P* for trend	<.001	<.001	<.001	<.001	<.001	<.001
Dietary magnesium intake	0.946 (0.782, 1.110) < 0.001	1.120 (0.963, 1.277) < 0.001	0.835 (0.655, 1.015) < 0.001	0.143 (0.090, 0.228) < 0.001	0.118 (0.072, 0.194) < 0.001	0.116 (0.060, 0.226) < 0.001
Q1	Reference	Reference	Reference	Reference	Reference	Reference
Q2	0.171 (0.100, 0.242) < 0.001	0.223 (0.156, 0.291) < 0.001	0.190 (0.129, 0.251) < 0.001	0.687 (0.588, 0.802) < 0.001	0.637 (0.542, 0.750) < 0.001	0.624 (0.521, 0.747) < 0.001
Q3	0.252 (0.182, 0.322) < 0.001	0.336 (0.270, 0.403) < 0.001	0.254 (0.191, 0.318) < 0.001	0.662 (0.566, 0.773) < 0.001	0.615 (0.522, 0.724) < 0.001	0.598 (0.494, 0.724) < 0.001
Q4	0.451 (0.382, 0.520) < 0.001	0.549 (0.483, 0.615) < 0.001	0.438 (0.366, 0.509) < 0.001	0.460 (0.388, 0.545) < 0.001	0.423 (0.354, 0.505) < 0.001	0.410 (0.325, 0.516) < 0.001
*P* for trend	<.001	<.001	<.001	<.001	<.001	<.001
Dietary sodium intake (g)	0.129 (0.116, 0.142) < 0.001	0.119 (0.106, 0.132) < 0.001	0.062 (0.048, 0.075) < 0.001	0.772 (0.741, 0.804) < 0.001	0.800 (0.767, 0.835) < 0.001	0.848 (0.807, 0.892) < 0.001
Q1	Reference	Reference	Reference	Reference	Reference	Reference
Q2	0.251 (0.181, 0.321) < 0.001	0.250 (0.183, 0.317) < 0.001	0.158 (0.098, 0.219) < 0.001	0.707 (0.609, 0.821) < 0.001	0.736 (0.629, 0.860) < 0.001	0.792 (0.668, 0.939) 0.007
Q3	0.383 (0.314, 0.452) < 0.001	0.373 (0.307, 0.440) < 0.001	0.203 (0.141, 0.265) < 0.001	0.578 (0.494, 0.675) < 0.001	0.629 (0.534, 0.740) < 0.001	0.746 (0.621, 0.896) 0.002
Q4	0.678 (0.610, 0.747) < 0.001	0.631 (0.565, 0.697) < 0.001	0.343 (0.276, 0.410) < 0.001	0.321 (0.268, 0.385) < 0.001	0.382 (0.316, 0.461) < 0.001	0.503 (0.402, 0.629) < 0.001
*P* for trend	<.001	<.001	<.001	<.001	<.001	<.001

Crude: no covariates were adjusted. Model 1: age, gender and race/ethnicity were adjusted. Model 2: age, gender, race/ethnicity, smoking behavior, educational level, BMI, PIR, serum uric acid, serum cholesterol, PRAL, dietary calcium intake, dietary Vitamin D intake.

BMD = bone mineral density, BMI = body mass index, PIR = poverty income ratio, PRAL = potential renal acid load.

**Table 3:** The association between dietary intake and the prevalence of osteoporosis.

	Crude OR (95% CI) *P* value	Model 1 OR (95% CI) *P* value	Model 2 OR (95% CI) *P* value
Dietary potassium intake (g)	0.629 (0.517, 0.765) < 0.001	0.617 (0.500, 0.761) < 0.001	0.644 (0.485, 0.856) 0.002
Q1	Reference	Reference	Reference
Q2	0.974 (0.628, 1.511) 0.907	0.891 (0.567, 1.401) 0.618	0.916 (0.558, 1.502) 0.727
Q3	0.411 (0.233, 0.725) 0.002	0.392 (0.219, 0.700) 0.002	0.407 (0.211, 0.783) 0.007
Q4	0.289 (0.151, 0.550) < 0.001	0.289 (0.149, 0.559) < 0.001	0.348 (0.152, 0.797) 0.013
*P* for trend	<.001	<.001	<.001
Dietary magnesium intake (g)	0.035 (0.006, 0.188) < 0.001	0.071 (0.012, 0.410) 0.003	0.334 (0.038, 2.952) 0.324
Q1	Reference	Reference	Reference
Q2	0.741 (0.474, 1.159) 0.189	0.777 (0.492, 1.227) 0.279	0.888 (0.537, 1.469) 0.644
Q3	0.407 (0.238, 0.697) 0.001	0.463 (0.267, 0.801) 0.006	0.500 (0.268, 0.933) 0.029
Q4	0.234 (0.121, 0.453) < 0.001	0.291 (0.148, 0.570) < 0.001	0.392 (0.175, 0.880) 0.023
*P* for trend	<.001	<.001	<.001
Dietary sodium intake (g)	0.649 (0.558, 0.754) < 0.001	0.750 (0.640, 0.878) < 0.001	0.842 (0.704, 1.007) 0.060
Q1	Reference	Reference	Reference
Q2	0.524 (0.328, 0.839) 0.007	0.625 (0.386, 1.010) 0.055	0.738 (0.444, 1.226) 0.241
Q3	0.427 (0.258, 0.705) < 0.001	0.609 (0.362, 1.022) 0.060	0.827 (0.468, 1.461) 0.513
Q4	0.193 (0.098, 0.381) < 0.001	0.362 (0.180, 0.730) 0.004	0.608 (0.278, 1.331) 0.213
*P* for trend	<.001	<.001	<.001

Crude: no covariates were adjusted. Model 1: age and race/ethnicity were adjusted. Model 2: age, race/ethnicity, smoking behavior, educational level, BMI, PIR, serum uric acid, serum cholesterol, PRAL, dietary calcium intake, dietary Vitamin D intake.

BMD = bone mineral density, BMI = body mass index, PIR = poverty income ratio, PRAL = potential renal acid load.

### 3.3. Stratified analysis of the relationship between multiple nutrients and BMD

**Figure 2.**
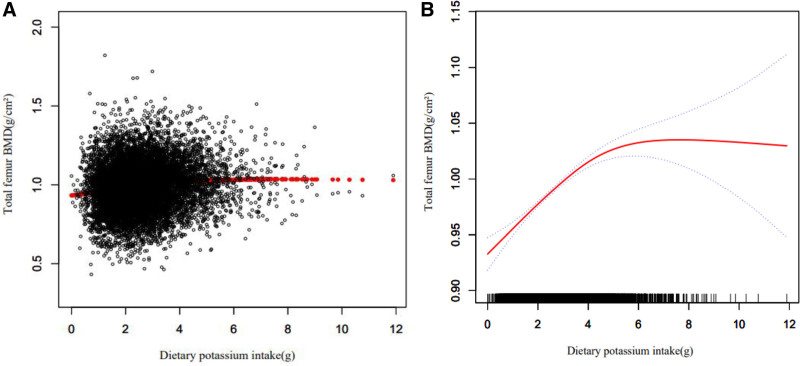
The association between dietary potassium intake and total femur bone mineral density (BMD). (A) Each black point represents a sample. (B) Red line represents the smooth curve fit between variables. Blue lines represent the 95% of confidence interval from the fit. Age, race/ethnicity, smoking behavior, educational level, body mass index, poverty income ratio, serum uric acid, serum cholesterol, potential renal acid load, dietary calcium intake, dietary vitamin D intake were adjusted.

**Figure 3.**
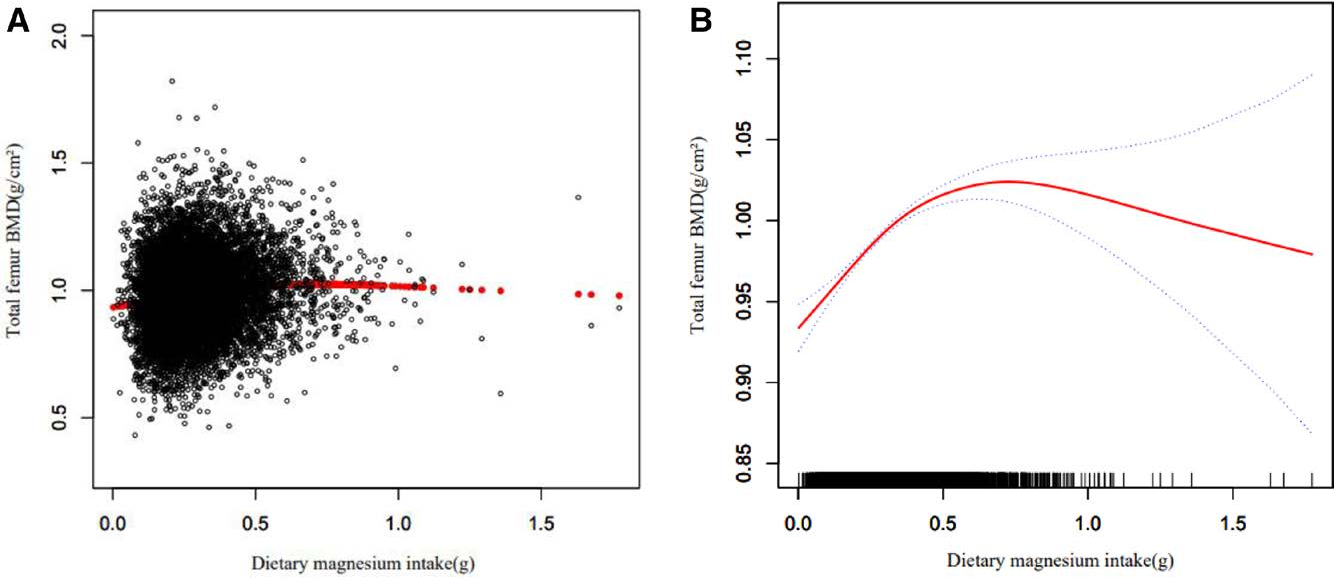
The association between dietary magnesium intake and total femur bone mineral density (BMD). (A) Each black point represents a sample. (B) Red line represents the smooth curve fit between variables. Blue lines represent the 95% of confidence interval from the fit. Age, race/ethnicity, smoking behavior, educational level, body mass index, poverty income ratio, serum uric acid, serum cholesterol, potential renal acid load, dietary calcium intake, dietary vitamin D intake were adjusted.

**Figure 4.**
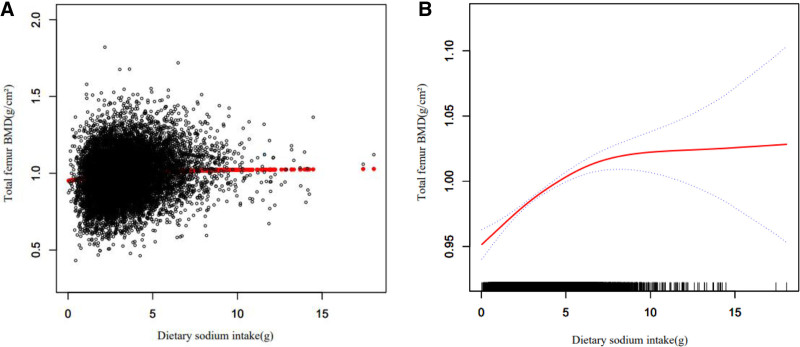
The association between dietary sodium intake and total femur bone mineral density (BMD). (A) Each black point represents a sample. (B) Red line represents the smooth curve fit between variables. Blue lines represent the 95% of confidence interval from the fit. Age, race/ethnicity, smoking behavior, educational level, body mass index, poverty income ratio, serum uric acid, serum cholesterol, potential renal acid load, dietary calcium intake, dietary vitamin D intake were adjusted.

Tables 4–6 show the relationship between multiple nutrient intake and whole femur BMD when analyzed by stratifying by sex, age, and ethnicity. Potassium ingestion had a positive correlation with whole femur BMD (β = 0.017 95% CI: 0.014–0.020, *P* < .001), but as shown in Table 4 among other Hispanics (β = 0.003 95% CI: -0.006 to 0.011, *P* = .503) and other races (including multi-racial) (β = 0.008 95% CI: -0.002 to 0.018, *P* = .124), potassium ingestion showed a nonsignificant relationship with whole femur BMD, and this positive correlation was not statistically significant. Magnesium intake correlated with whole femur BMD positively (β = 0.102 95% CI: 0.080–0.124, *P* < .001), but this positive correlation was not statistically significant among other Hispanics (β = 0.051 95% CI: -0.016 to 0.118, *P* = .138), as shown in Table 5.Sodium intake was positively associated with whole femur BMD (β = 0.002 95% CI: 0.000–0.003, *P* = .030), but as shown in Table 6 in other Hispanic (β = 0.051 95% CI: -0.001 to 0.010, *P* = .123), other races (including multi-racial) (β = 0.001 95% CI: -0.004 to 0.006, *P* = .566) and men (β = 0.001 95% CI: -0.001 to 0.003, *P* = .475) this positive correlation was not statistically significant. Figures 2–4 show the association between multiple nutrient intake and whole femur BMD by smooth curve fitting.

**Table 4:** The association between dietary potassium intake and femur BMD (g/cm²).

	Model 1 β (95% CI) *P* value	Model 2 β (95% CI) *P* value	Model 3 β (95% CI) *P* value
Dietary potassium intake (g)	0.014 (0.012, 0.017) < 0.001	0.018 (0.016, 0.021) < 0.001	0.017 (0.014, 0.020) < 0.001
Dietary potassium intake (g) categories			
Q1 (≤0.995)	Reference	Reference	Reference
Q2 (0.996–1.319)	0.006 (-0.003, 0.015) 0.174	0.016 (0.008, 0.025) < 0.001	0.014 (0.006, 0.021) < 0.001
Q3 (1.320–1.760)	0.019 (0.010, 0.027) < 0.001	0.036 (0.028, 0.044) < 0.001	0.028 (0.021, 0.036) < 0.001
Q4 (≥1.761)	0.044 (0.035, 0.052) < 0.001	0.060 (0.052, 0.068) < 0.001	0.051 (0.042, 0.060) < 0.001
*P* for trend	<.001	<.001	<.001
Subgroup analysis stratified by gender			
Male	0.003 (-0.000, 0.006) 0.055	0.006 (0.003, 0.009) < 0.001	0.005 (0.002, 0.009) 0.003
Female	-0.000 (-0.004, 0.004) 0.930	0.008 (0.004, 0.011) < 0.001	0.013 (0.009, 0.018) < 0.001
Subgroup analysis stratified by race/ethnicity			
Mexican American	0.018 (0.013, 0.023) < 0.001	0.018 (0.013, 0.023) < 0.001	0.016 (0.011, 0.022) < 0.001
Other Hispanic	0.003 (-0.004, 0.010) 0.423	0.005 (-0.002, 0.012) 0.158	0.003 (-0.006, 0.011) 0.503
Non-Hispanic White	0.018 (0.015, 0.022) < 0.001	0.020 (0.017, 0.023) < 0.001	0.019 (0.015, 0.023) < 0.001
Non-Hispanic Black	0.017 (0.011, 0.023) < 0.001	0.016 (0.010, 0.022) < 0.001	0.016 (0.008, 0.023) < 0.001
Other races (including multi-racial)	0.014 (0.004, 0.023) 0.004	0.016 (0.007, 0.025) < 0.001	0.008 (-0.002, 0.018) 0.124
Subgroup analysis stratified by age			
20–34	0.012 (0.008, 0.016) < 0.001	0.015 (0.010, 0.019) < 0.001	0.013 (0.008, 0.018) < 0.001
35–49	0.013 (0.009, 0.017) < 0.001	0.016 (0.012, 0.020) < 0.001	0.014 (0.010, 0.019) < 0.001
≥50	0.022 (0.017, 0.026) < 0.001	0.023 (0.019, 0.027) < 0.001	0.023 (0.018, 0.028) < 0.001

Crude: no covariates were adjusted. Model 1: age and race/ethnicity were adjusted. Model 2: age, race/ethnicity, smoking behavior, educational level, BMI, PIR, serum uric acid, serum cholesterol, PRAL, dietary calcium intake, dietary Vitamin D intake.

BMD = bone mineral density, BMI = body mass index, PIR = poverty income ratio, PRAL = potential renal acid load.

**Table 5:** The association between dietary magnesium intake and femur BMD (g/cm²).

	Model 1 β (95% CI) *P* value	Model 2 β (95% CI) *P* value	Model 3 β (95% CI) *P* value
Dietary magnesium intake (g)	0.115 (0.095, 0.135) < 0.001	0.137 (0.118, 0.156) < 0.001	0.102 (0.080, 0.124) < 0.001
Dietary magnesium intake (g)categories			
Q1 (≤0.995)	Reference	Reference	Reference
Q2 (0.996–1.319)	0.021 (0.012, 0.029) < 0.001	0.027 (0.019, 0.035) < 0.001	0.023 (0.016, 0.031) < 0.001
Q3 (1.320–1.760)	0.031 (0.022, 0.039) < 0.001	0.041 (0.033, 0.049) < 0.001	0.031 (0.023, 0.039) < 0.001
Q4 (≥1.761)	0.055 (0.047, 0.063) < 0.001	0.067 (0.059, 0.075) < 0.001	0.053 (0.045, 0.062) < 0.001
*P* for trend	<.001	<.001	<.001
Subgroup analysis stratified by gender			
Male	0.039 (0.014, 0.064) 0.002	0.056 (0.032, 0.080) < 0.001	0.056 (0.027, 0.084) < 0.001
Female	0.004 (-0.027, 0.035) 0.800	0.042 (0.013, 0.070) 0.004	0.047 (0.015, 0.079) 0.0041
Subgroup analysis stratified by race/ethnicity			
Mexican American	0.165 (0.124, 0.206) < 0.001	0.171 (0.132, 0.211) < 0.001	0.121 (0.075, 0.167) < 0.001
Other Hispanic	0.040 (-0.021, 0.102) 0.197	0.050 (-0.008, 0.108) 0.094	0.051 (-0.016, 0.118) 0.138
Non-Hispanic White	0.137 (0.108, 0.167) < 0.001	0.132 (0.104, 0.160) < 0.001	0.095 (0.063, 0.128) < 0.001
Non-Hispanic Black	0.141 (0.086, 0.197) < 0.001	0.137 (0.083, 0.190) < 0.001	0.100 (0.037, 0.162) 0.002
Other races (including multi-racial)	0.203 (0.124, 0.282) < 0.001	0.221 (0.144, 0.298) < 0.001	0.186 (0.096, 0.276) < 0.001
Subgroup analysis stratified by age			
20–34	0.068 (0.033, 0.104) < 0.001	0.094 (0.059, 0.129) < 0.001	0.049 (0.006, 0.092) 0.025
35–49	0.115 (0.084, 0.146) < 0.001	0.136 (0.105, 0.167) < 0.001	0.113 (0.078, 0.149) < 0.001
≥50	0.164 (0.130, 0.199) < 0.001	0.160 (0.126, 0.194) < 0.001	0.118 (0.080, 0.156) < 0.001

Crude: no covariates were adjusted. Model 1: age and race/ethnicity were adjusted. Model 2: age, race/ethnicity, smoking behavior, educational level, BMI, PIR, serum uric acid, serum cholesterol, PRAL, dietary calcium intake, dietary Vitamin D intake.

BMD = bone mineral density, BMI = body mass index, PIR = poverty income ratio, PRAL = potential renal acid load.

**Table 6:** The association between dietary sodium intake and femur BMD (g/cm²).

	Model 1 β (95% CI) *P* value	Model 2 β (95% CI) *P* value	Model 3 β (95% CI) *P* value
Dietary sodium intake (g)	0.016 (0.014, 0.017) < 0.001	0.005 (0.003, 0.007) < 0.001	0.002 (0.000, 0.003) 0.030
Dietary potassium intake (g)categories			
Q1 (≤0.995)	Reference	Reference	Reference
Q2 (0.996–1.319)	0.031 (0.022, 0.039) < 0.001	0.030 (0.022, 0.039) < 0.001	0.019 (0.012, 0.027) < 0.001
Q3 (1.320–1.760)	0.047 (0.038, 0.055) < 0.001	0.046 (0.037, 0.054) < 0.001	0.025 (0.017, 0.032) < 0.001
Q4 (≥1.761)	0.083 (0.074, 0.091) < 0.001	0.077 (0.069, 0.085) < 0.001	0.042 (0.034, 0.050) < 0.001
*P* for trend	<.001	<.001	.002
Subgroup analysis stratified by gender			
Male	0.005 (0.003, 0.006) < 0.001	0.004 (0.002, 0.006) < 0.001	0.001 (-0.001, 0.003) 0.475
Female	0.010 (0.008, 0.013) < 0.001	0.008 (0.006, 0.011) < 0.001	0.005 (0.002, 0.007) < 0.001
Subgroup analysis stratified by race/ethnicity			
Mexican American	0.014 (0.010, 0.017) < 0.001	0.012 (0.009, 0.016) < 0.001	0.006 (0.003, 0.010) < 0.001
Other Hispanic	0.013 (0.008, 0.019) < 0.001	0.009 (0.004, 0.015) < 0.001	0.005 (-0.001, 0.010) 0.123
Non-Hispanic White	0.019 (0.016, 0.021) < 0.001	0.016 (0.014, 0.018) < 0.001	0.008 (0.006, 0.011) < 0.001
Non-Hispanic Black	0.016 (0.012, 0.020) < 0.001	0.013 (0.009, 0.017) < 0.001	0.010 (0.006, 0.014) < 0.001
Other races (including multi-racial)	0.010 (0.005, 0.016) < 0.001	0.009 (0.004, 0.014) 0.001	0.001 (-0.004, 0.006) 0.566
Subgroup analysis stratified by age			
20–34	0.013 (0.009, 0.017) < 0.001	0.016 (0.012, 0.020) < 0.001	0.014 (0.010, 0.019) < 0.001
35–49	0.010 (0.008, 0.013) < 0.001	0.011 (0.009, 0.014) < 0.001	0.003 (0.001, 0.006) 0.012
≥50	0.020 (0.017, 0.023) < 0.001	0.018 (0.015, 0.021) < 0.001	0.009 (0.007, 0.012) < 0.001

Crude: no covariates were adjusted. Model 1: age and race/ethnicity were adjusted. Model 2: age, race/ethnicity, smoking behavior, educational level, BMI, PIR, serum uric acid, serum cholesterol, PRAL, dietary calcium intake, dietary Vitamin D intake.

BMD = bone mineral density, BMI = body mass index, PIR = poverty income ratio, PRAL = potential renal acid load.

## 4. Discussion

The results of this study, based on data from a large NHANES sample, demonstrate an association between intake of multiple dietary nutrients (potassium, magnesium, and sodium) and lumbar spine bone density. We found that intake of dietary alkalizing elements (potassium, sodium, and magnesium) is correlated positively with BMD and adversely with the incidence of low BMD in the U.S. population, whereas potassium intake remains adversely correlated with the incidence of osteoporosis.

A previous cross-sectional study investigated the relationship between nutrient intake and BMD in 243 premenopausal (29–60 years) and 137 postmenopausal (39–60 years) Japanese women and found that only postmenopausal women had a significant positive relationship between potassium intake and BMD.^[16]^ In 2010, Hattie H. Wright et al found that dietary magnesium intake predicted bone turnover in humans in a prospective study of postmenopausal Black urban South African women.^[17]^ A cross-sectional study from Australia investigated the relationship between nutrient inclusion and BMD in prepubertal children and found a negative or no correlation between dietary potassium and magnesium intake and BMD.^[15]^ Another cross-sectional analysis of 57 men aged 39 to 42 years from Canada concluded that an abundance of potassium and phosphorus was beneficial in maintaining BMD in adult men.^[8]^ Our study found that dietary alkaline element (potassium, sodium, magnesium) intake was positively correlated with BMD. This result can be explained in several ways. Firstly, the research by Susan J. Whiting et al only included 39 to 42 year old males in a cross-sectional analysis and did not analyze females. Second, the study did not eliminate the effect of covariates on the results. Third, the study had a limited number of participants, and despite the weighted analyses, it does not provide an accurate representation of the whole U.S. population which might have skewed the findings.

We demonstrated a significant positive association of dietary potassium, sodium, and magnesium intake with BMD in this study. We believe a number of underlying processes could provide an explanation for this finding. The first and most likely theory is the acid–base mechanism.^[18]^ Acid loading may buffer the alkaline calcium salts in the bones, thus leading to bone loss.^[19]^ Acidic conditions promote increased osteoclast activity and decreased osteoblast activity.^[20,21]^ Potassium, sodium, and magnesium, which are metallic elements, are oxidized in the body to produce alkaline oxides such as K_2_O, Na_2_O, and MgO.^[22]^ As buffer bases, they neutralize excess acid in the body and eliminate the negative effects of acid on bone mass. A survey from Canada also concluded that an alkaline diet would prevent bone loss and that a high acid diet would lead to bone loss.^[23]^ Secondly, several researches have shown that there is a strong link between calcium levels in the body and BMD, and that dietary alkaline intake may also improve calcium balance by reducing urinary calcium excretion.^[24,25]^ In a previous short-term study, the use of 50 mmol of potassium bicarbonate was found to reduce acute urinary calcium excretion in adults.^[26]^ Therefore, dietary potassium may influence bone resorption by affecting calcium homeostasis. Third, food sources rich in potassium and magnesium are often rich in other nutrients that are beneficial to bones, such as calcium, Vitamin D, and protein. One study concluded that potassium and magnesium intake is a sign of a high-quality diet.^[14]^ In addition, previous studies have shown that lower intakes of potassium and magnesium are related to a higher rate of osteoconversion, which is considered to be an essential determination of osteoporosis or fragility fracture.^[27]^ Although the associations were postulated and discovered, the precise underlying principles of the positive associations between dietary alkalizing elements and BMD are unknown and need to be thoroughly discussed, investigated, and validated.

In addition, studies have shown that positive associations among dietary intakes of potassium, sodium, and magnesium and BMD tend to vary by gender, age, and ethnic group. Within the context of what we did in this research, we observed no statistically significant positive correlation among potassium, sodium, and magnesium intake and BMD in other Hispanics. Differences in genetics, lifestyle, and other factors may explain the racial differences in this relationship. Further research is needed to provide strong evidence for sex, age, and race differences in this relationship.

Our study has the following advantages. First, it is a large multiethnic demographic analysis based on the NHANES survey, and our weighted analysis improves the reliability and standardization of the data to make it more representative of the general U.S. population. On the contrary, in order to accurately analyze the positive correlation between dietary potassium, magnesium, and sodium intake and BMD, we rigorously screened and adapted a wide range of confounding factors affecting BMD. In addition, we also analyzed the association between dietary potassium, magnesium, and sodium intake and low BMD and osteoporosis. As far as we know, it is an initial effort to analyze the association among dietary potassium, magnesium, and sodium intake and osteoporosis using data from NHANES (2005–2010, 2013–2014, and 2017–2018).

Of course, our study has several limits. First, since our research was a cross-sectional study, it was not possible to investigate the cause-and-effect relationship between dietary intake of potassium, sodium, and magnesium and BMD. Secondary, incomplete data prevented us from exploring the association of dietary intake of potassium, sodium, and magnesium with BMD in the rest of the torso. Correlative studies have shown that the association among dietary potassium, sodium, and magnesium intake and BMD may vary among various bone locations.^[15]^ Finally, it is possible that we did not account for the influence of related confounding factors, like other dietary intake, occupation, estrogen status, and physical activity habits, which could have led to errors.

## 5. Conclusions

Our findings suggest that dietary potassium, magnesium, and sodium intakes are positively associated with BMD in the U.S. population, and that confounding factors such as sex, age, and race may influence this relationship. The association of dietary potassium, magnesium, and sodium intake with BMD, low BMD, and osteoporosis requires not only in-depth research on the basis of a substantial body of research evidence, but also future exploration of the specific mechanisms and confounding factors involved.

## Acknowledgments

We acknowledge the data from the National Health and Nutrition Examination Survey (NHANES).

## Author contributions

**Conceptualization:** Xiaotong Feng.

**Data curation:** Wenfeng Hu, Xiaotong Feng, Chaoqun Wen.

**Methodology:** Chaoqun Wen.

**Software:** Wenfeng Hu, Xiaotong Feng.

**Validation:** Wenfeng Hu.

**Visualization:** Wenfeng Hu.

**Writing – original draft:** Wenfeng Hu, Chaoqun Wen.

**Writing – review & editing:** Xiaotong Feng, Chaoqun Wen.
